# The Effects of COVID-19 Risk Perception on Travel Intention: Evidence From Chinese Travelers

**DOI:** 10.3389/fpsyg.2021.655860

**Published:** 2021-07-16

**Authors:** Yue Meng, Asif Khan, Sughra Bibi, Haoyue Wu, Yao Lee, Wenkuan Chen

**Affiliations:** ^1^College of Tourism, Sichuan Agricultural University, Chengdu, China; ^2^Department of Tourism and Hotel Management, School of Management, Zhejiang University, Hangzhou, China; ^3^Department of Tourism and Hospitality, Hazara University, Mansehra, Pakistan; ^4^Guanghua Law School Zhejiang University, Hangzhou, China

**Keywords:** COVID-19, risk perception, risk knowledge, travel intention, interpersonal and media communication, demographic influence

## Abstract

This study attempts to assess the relationship between risk perception, risk knowledge, and travel intentions of Chinese leisure travelers during the COVID-19 pandemic in the framework of social contagion and risk communication theories by analyzing a sample of 1,209 travelers through structural equation modeling (SEM) and path analysis. We used the process macro of Hayes to analyze the moderation effects of age, gender, and education between risk perception, media and interpersonal communication, and risk knowledge. It was found that travelers were more concerned about self-efficacy than severity. Risk perception of travelers predicts the information-seeking process of tourists. This process helps travelers to accumulate risk information that influences their travel intentions. Travelers give more importance to interpersonal (contagion) communication in making a traveling decision. Demographic factors influence traveling decision-making; women travelers were found to be more risk resilient than men. Young travelers seek information at low- and old travelers at high-risk levels. Marketing implications also provided.

## Introduction

The tourism industry is most vulnerable to natural disasters, conflicts, terrorism, and economic crisis. The health measures and communication approaches, such as homestay campaigns, lockdowns, travel bans, quarantine, and social distancing, have ceased tourism-related industries operations. The tourism industry shows its resilience in bouncing back from major economic, political, and health crises (Sigala, 2020); however, the unprecedented vulnerabilities of COVID-19 unveiled that the crisis is different and would have long-lasting structural changes to the tourism industry. The COVID-19 pandemic has challenged the existing economic and tourism systems, has led the world to a recession, and has limited the potential of travelers to their homes. The COVID-19 epidemic undoubtedly uncovered that the lack of knowledge restrained the capability of the tourism industry to manage the uncertainty and risk of this magnitude.

The Chinese outbound tourism market becomes an attention point for the international tourism industry to boost their economies (Yu et al., 2020). Outbound Chinese travelers have become a source of earnings for millions of people in the rest of the world (Wen et al., 2020). Since the start of the new Lunar year, travel agencies and airlines in China have suspended their operations. In the Spring Festival, millions of Chinese travelers usually travel across the country and abroad; however, in response to COVID-19, all the traveling has been suspended (Bogoch et al., 2020). The significance of Chinese travelers to the world makes it of considerable relevance to examine and understand their psychological and behavioral drives and their reaction to travel post-COVID-19.

The tourism industry of China is multiplying and becoming a significant part of the Chinese economy (Li et al., 2010). The domestic and outbound travel boom in China is due to the emergence of an affluent middle class and to ease of movement (Huang et al., 2015). Over the last few decades, since the beginning of the reforms and open-door policy, China has become the busiest outbound and inbound tourist market (Shambaugh, 2013). It was estimated that the number of domestic trips in China would increase to about 2.38 billion trips by 2020 (Rosen, 2018). “China is the single largest outbound travel market in the world in terms of spending” (Ying et al., 2020). The major factors driving the growth of the outbound tourism market of China include a rising affluent middle-class population, a liberal tourism policy, and an open-door policy. We choose China for the present study because it is among the top 10 global destinations, and, when it comes to outbound tourism, China leads the way in terms of total spending worldwide (UNWTO, 2018). Chinese tourists made 150 million outbound trips in 2018 and spent $227 billion (UNWTO, 2019); however, due to COVID-19, a total of 25 million outbound trips are estimated this year that could wipe out $73 billion spending (Folinas and Metaxas, 2020). As the Lunar New Year of China begins, under normal circumstances, ~400 million Chinese travelers make 3 billion trips across China, out of which 7 million were estimated to travel abroad (Reuters, 2019); however, COVID-19 ceased this massive migration in 2020.

The growing discussion on the tourism industry and COVID-19 pandemic calls for a deeper understanding of traveler risk and intention to travel (Khan et al., 2020b,e). The cognizance of travel risk formation of perceived COVID-19, risk knowledge, and willingness to act according to the outbreak and behavioral changes would enable the industry stakeholders to recover and reform the existing norms. The transformation of the tourism industry depends on the behavior of the travelers in response to a potential crisis (Sigala, 2020). There is an extensive stream of knowledge about tourism, terrorism risk, and political risk; however, there are few studies on tourism and health risks, such as those of Jonas et al. (2011), and Wang et al. (2019). Hence, these studies are conducted in a normal situation, overlooking the severity of a pandemic like COVID-19 on traveler psychological condition and behavioral intentions. The literature on the perceived risk of infectious diseases, such as SARS, HINI, and Ebola, comes from the studies, for instance, of Kim et al. (2015), Gee and Skovdal (2017), and Brug et al. (2004). These studies are mostly descriptive and are not based on firm theoretical backgrounds; besides, the validity and the reliability of the scales are unconfirmed. However, this study is based on a solid theoretical background and provided a reliable and validated measure of COVID-19 risk perception and its connection to media channels, risk knowledge building, and travel intentions, which make our study unique.

The purpose of this research is to examine travel risk perception and travel intentions (Chinese travelers) with relevant elements by applying contagion and risk communication theories in the context of COVID-19 to understand traveler risk behavior. Specifically, this research investigates the relative importance of media and peer groups in reshaping health risk knowledge that influences travel behavior, taking into account the demographic factors that influence the behavior of the travelers. This study is also motivated by the call of the scholars to investigate the behavioral response of the Chinese travelers to COVID-19. Travel decisions are complicated and risky; travelers are always encouraged to search for new information (Griffin et al., 2004), and, nowadays, this is done by relying on media information and social network opinions (Leder et al., 2015).

To do so, we collected data through an online survey, targeting leisure travelers, living in various regions of China, who visited a foreign country at least one time within the past 3–5 years. The survey was available for completion between June 2, 2020, and August 29, 2020. The final sample includes 1,209 filled questionnaires. An exploratory analysis was performed for the initial reliability of measurements, followed by confirmatory factor analysis to confirm the validity of the scales. We applied structural equation modeling and path analysis for analyzing the relationship between the variables. It is expected that the outcomes of this research would enlarge the understanding of risk perception and travel intentions of travelers during COVID-19; besides, it would inform the industry, policymakers, and stakeholders about reshaping the current value system, health priorities, and advancement in technology.

## Theoretical Background and Hypotheses

The risk associated with the COVID-19 pandemic is expected to have far reaching influence on the travel intentions. These influences would vary from person to person, having different sociocultural backgrounds. This study focuses on Chinese potential leisure travelers to debate the impacts of COVID-19 on their travel intentions. With the risk of human-to-human spread of COVID-19, Chinese authorities passed policies for social distancing and avoidance to travel (Chen et al., 2020). People have lost their traditional lifestyle due to the fear of COVID-19, for instance, virtual buying, entertainment, and travel experience (Sigala, 2020). The COVID-19 earlier tourism research mostly focused on the economic impacts and survival; however, less attention is paid to travel behaviors, intentions to inform businesses when to resume operations, and what segments of the market to target (Gössling et al., 2020). It is essential to investigate the basic unit (traveler) of the tourism industry for transformation and inform all the stakeholders what strategies and new ways would benefit the future.

Risk is an inherent segment of traveling decision-making for (international) travelers (Reisinger and Mavondo, 2005). In the tourism literature, perceived risk includes the feeling of fear, nervousness, anxiety, and worry (Reisinger and Mavondo, 2005; Rittichainuwat and Chakraborty, 2009) or the perceived probability of (very) bad events (Ritchie et al., 2017). Thus, travel-related decision-making is complicated (Quintal et al., 2010); this motivates travelers to get more information to manage risk and uncertainty. Risk information-seeking behavior enhances traveler risk knowledge about the travel destination and ultimately influences traveling intentions (Griffin et al., 1999). Travelers improve their risk knowledge by obtaining information either from a social network or from mass media. Research indicates that risk communication and risk perception collectively impact traveler behavioral intentions (Leder et al., 2015). Risk communication aims to inform people who are threatened by the perceived risk (Leder et al., 2015). Travelers integrate this broad set of information and network opinions into their traveling decisions.

**Figure 1:**
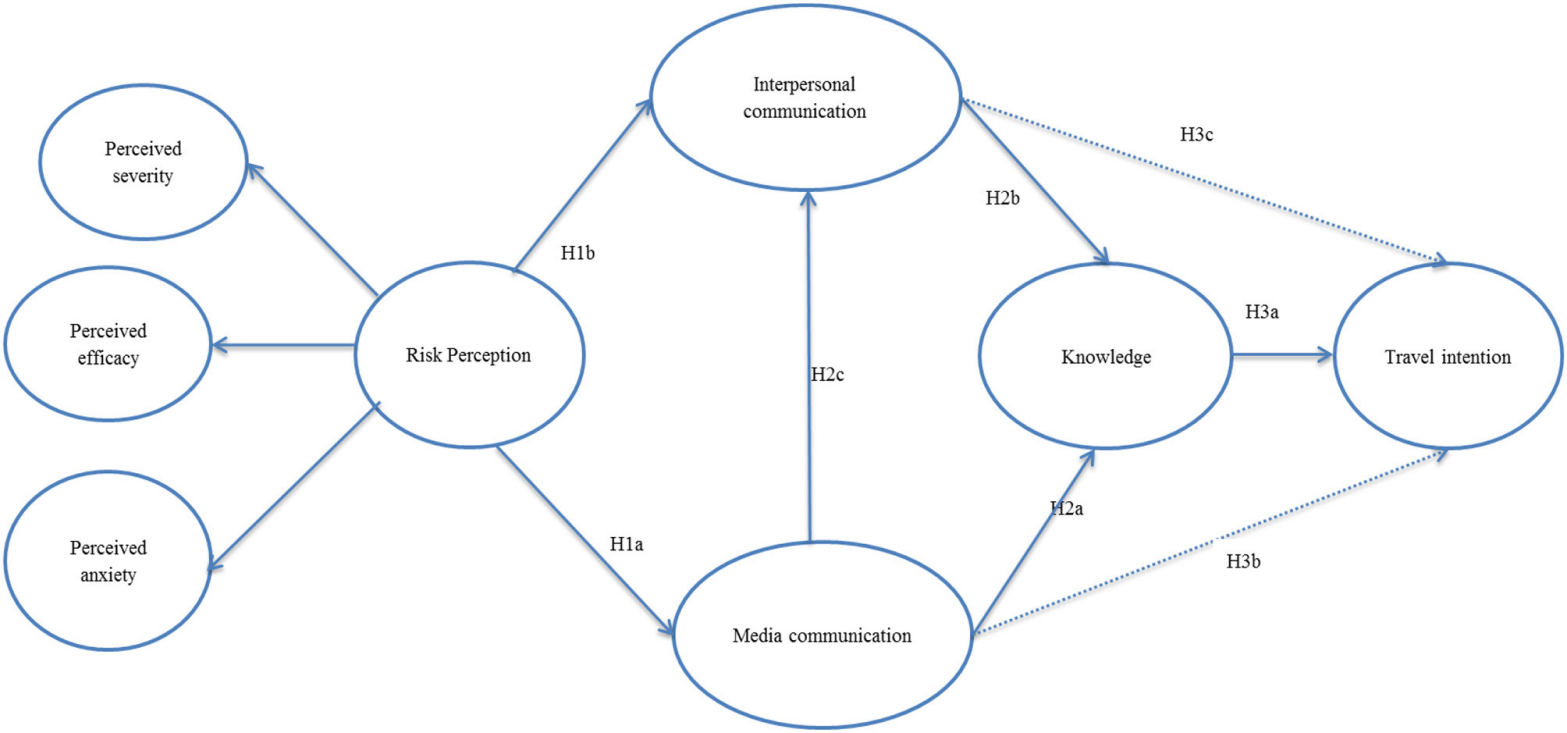
Conceptual modeling.

Social contagion theory explains the underlying mechanism of how an individual level of communication influences risk knowledge (Muter et al., 2013). “The idea of social contagion poses that individuals adopt the attitudes or behaviors of others in the social network with whom they communicate. The theory does not require that there is intent to influence, or even an awareness of influence, only that communication takes place” (Scherer and Cho, 2003). Social networks function as critical channels in which people receive, share, and exchange information about risk-related events. Although social networks are recognized as essential sources of social influence, no such study exists in tourism literature that explicitly explored the impact of risk perception contagion on risk knowledge and travel intentions. The conceptual path model of the research is presented in Figure 1 (additional supporting materials are provided in Supplementary Material).

### Risk Perception

Risk is the subjective feeling of an individual concerning uncertainty (Quintal et al., 2010). Perceived risk has been conceptualized as the subjective determinant of expected potential losses, where each outcome has assigned a probability (Dholakia, 2001). In tourism literature, perceived risk has been identified as a multifaceted phenomenon comprised of several risk factors (Chien et al., 2017). Tourists avoid traveling when the perceived health risk is high (Aliperti and Cruz, 2018; Khan et al., 2020a, 2021). Perceived risk has been measured as a combination of perceived severity (magnitude), anxiety (feeling of worry, nervousness), and efficacy (safety concerns) (Reisinger and Mavondo, 2005; Rittichainuwat and Chakraborty, 2009). Scholars used various measures during SARS and HINI outbreaks to map the risk perception (Leppin and Aro, 2009; Bults et al., 2011; Kim et al., 2015). Anxiety is considered a salient factor in assessing perceived risk (Davis-Berman and Berman, 2002). Severity is a critical determinant of predicting risk perception (Brewer et al., 2004). This discussion leads us to assume that severity, efficacy, and anxiety lead to risk perception (additional supporting materials are provided in Supplementary Material).

### Risk Perception, Media Communication, Interpersonal Communication, Knowledge, and Travel Intention

Travelers are easy victims of infectious diseases (Baker, 2015). Tourists often experience a great degree of severity, anxiety, and efficacy to epidemic and pandemic outbreaks when traveling internationally (Korstanje, 2011; Khan et al., 2020c,f). Perceived risk is viewed as a motivational factor that influences subsequent travel intentions, knowledge searches, and dissemination of information, and visiting decision-making (Dholakia, 2001). Travelers search for information to reduce the degree of risk associated with their travel (Atkin and Thach, 2012). Risk perception is regarded as the antecedent of risk information-seeking behavior (Huurne and Gutteling, 2008). Mass media provide the audience with relevant information about risk (Hall, 2002). Additional supporting materials are provided in Supplementary Material under the title “Risk Perception of COVID-19 and Travel Intention.” Thus, we assume that COVID-19 risk perception has an association with media and interpersonal communication.

H1a: The COVID-19 risk perception influences traveler information-seeking behavior from a mass media communication.

H1b: The COVID-19 risk perception influences traveler information-seeking behavior from interpersonal communication.

Most of the studies have examined that audiences use mass media during the outbreak of infectious disease to enhance their level of risk knowledge (Pandey et al., 2010; Khan et al., 2020e). Individuals actively seek information when making an important decision about their health (Huurne and Gutteling, 2008). The increase in risk boosts the desire to seek information to improve individual risk knowledge and guide their traveling decisions (Huurne and Gutteling, 2008). The Risk Information Seeking and Processing (RISP) model (Yang et al., 2014), the heuristic-systematic model (HSM) (Chaiken, 1999), the theory of planned behavior (TPB) (Ajzen, 1991), and the model of the Information Search Process (IPS) (Kuhlthau, 1991) allow an individual to investigate the critical drives of risk information. The individual social environment also increases the desire to gain more knowledge by seeking information. Decisions of individuals are greatly influenced by their family, friends, and a circle of colleagues (Ho, 2012). Thus, the following hypotheses are assumed.

H2a: Media communication affects traveler risk knowledge.

H2b: Interpersonal communication affects traveler risk knowledge.

H2c: Media communication has an association with interpersonal communication.

The growing amount of information accessible through mass media and the Internet can promote individual risk-avoiding behaviors (Stryker, 2003). Media information influencing individual behavior is greater if those individuals talk about media contents in interpersonal networks (Lee, 2009). Individuals are inclined to base their decisions first by considering what their social network thinks about the prevailing risk. This whole discussion leads us to conclude that individual travelers perceive a certain degree of risk about infectious diseases when intending to travel. The risk perception motivates travelers to seek more information to enhance their knowledge to reduce the risk of contagious diseases. The information flow either comes from mass media or interpersonal networks. At the same time, media communication has a positive association with interpersonal communication. This information-seeking behavior ultimately influences travel intention. Thus, we assume the following hypotheses:

H3a: Risk knowledge influences travel behavior intentions.

H3b: Media communication influences travel behavior intentions.

H3c: Interpersonal communication influences travel behavior intentions.

Demographic factors play a vital role in moderating the relationship between perceived risk and dependent variables (Kusumi et al., 2017). This discussion leads us to pose the following hypotheses:

H4a: Demographic factors (gender, age, and education) moderate the relationship between COVID-19 risk perception and interpersonal communication.

H4b: Demographic factors (gender, age, and education) moderate the relationship between COVID-19 risk perception and media communication.

H4c: Demographic factors (gender, age, and education) moderate the relationship between interpersonal communication and risk knowledge.

H4d: Demographic factors (gender, age, and education) moderate the relationship between media communication and risk knowledge.

## Research Method

A snowball sampling technique was used for the collection of data. We target only leisure travelers who visited a foreign country at least one time within the past 3–5 years. An online survey link was distributed through WeChat, Sina Weibo, and Tencent QQ in various regions of China with the reward of a red packet (minimum 10 yuan per participant) for the encouragement of the participants. It is ensured to receive a maximum response from the selected six regions, and candidates were recruited for conducting the survey. The survey was conducted from June 2, 2020 to August 29, 2020. We got a total of 1,209 polls; due to the precise nature of the survey based on the online link, we found no problem with missing data. The demographic characteristics indicate that, out of 1,209 participants, 58.8% were men and 45.2% were women. Besides, 47.3% belonged to 26–30 ys, 37.8% belonged to the salary group of 11,000–20,000 Yuans, and 53.2% were single. The results of travel intentions within 6 months after the pandemic revealed that ~56% of men and 66% of women would like to travel (for more details, see Table 1).

**Table 1:** Demographics profile.

**Characteristics**	**Frequency**	**Percentage**	**Characteristics**		**Frequency**	**Percentage**
**Gender**			**Income**			
Male	662	54.8	5000–10000		284	23.5
Female	547	45.2	11000–20000		457	37.8
**Education**			21000–30000		287	23.7
High School	359	29.7	31000 and above		181	15.0
Under-Graduate	581	48.1	**Marital status**			
Master and above	269	22.2	Married		566	46.8
**Age**			Single		643	53.2
15–20 years	9	0.70	Intention to travel			
21–25 years	358	29.6	Male	Yes	374	56.49
26–30 years	572	47.3		No	288	43.51
31–40 years	114	9.4	Female	Yes	365	66.73
41–50 years	86	7.1		No	182	33.27
51 and above years	70	5.8				
**Region**						
Beijing	210	17.4				
Shanghai	220	18.2				
Hubei	305	23.2				
Guangdong	154	12.7				
Zhejiang	200	16.5				
Jiangxi	150	9.93				

We also performed a normality test, suggesting that all our items have univariate normality. The skewness for all the items is <3, and kurtosis is <7. Our data failed to exhibit multivariate normality; however, it is not required for SEM. The SEM does not assume normality. Rresearchers estimate the parameters and assess the model using the maximum likelihood (ML) approach under some degree of multivariate non-normality. ML SEM can produce consistent parameter estimates even in the sense of non-normality (Wooldridge, 2009). Byrne adopted a kurtosis value of >7, indicating a departure from normality (West et al., 1995). Kline (2015) suggested values greater than 3 (in absolute value), which might indicate more extreme skew levels. If the univariate distributions are nonnormal, then the multivariate distribution will be nonnormal (West et al., 1995).

### Instrument Measurements

This conceptual model of the study is comprised of one exogenous and four endogenous variables, whereas perceived severity, efficacy, and anxiety form the risk perception variable. Interpersonal communication, media communication, knowledge, and traveling intentions are endogenous variables. This study deals with risk perception as a second-order construct. The scales for all measurements are adopted from the previous literature with maximum changes as per the requirement of COVID-19. The scales of perceived severity, efficacy, and anxiety are taken from Brug et al. (2004), Bults et al. (2011), and Lau et al. (2003). The perceived severity scale comprises four items: perceived efficacy consists of six items, and perceived anxiety consists of three items. The media communication and interpersonal communication scale contains eight items, each taken from the past studies of Gao et al. (2019) and Gee and Skovdal (2017). The knowledge scale consists of eight items in total, measuring the knowledge of the participants about the COVID-19 pandemic. The scale is taken from Brug et al. (2004) and Bults et al. (2011). The traveling behavior intention scale consists of seven items, measuring participant travel behavioral intentions during the pandemic. The scale is taken from Desivilya et al. (2015) and Schroeder et al. (2013). All the research items are designed on a 5-point Likert, ranging from 1 = strongly disagree to 5 = strongly agree. A list of all the research items is provided in the Supplementary Material Appendix AI.

## Results of Performed Analyses

### Common Method Variance

The data collected from the same source simultaneously in a cross-sectional design always pose a chance of common method variance (CMV) (Lindell and Whitney, 2001). In social science, it was found that CMV influences the outcomes; hence, it is recommended to control this issue (Podsakoff et al., 2012). Various techniques for assessing CMV in the dataset have been proposed, for instance, Harman's test (Chang et al., 2010). The results indicated that the total seven-factor solution explained 81.97% variance. We ran an EFA with a principal component with 22 items to examine the variance explained by a single factor. The single factor explained only a 46.54% variance out of the total; thus, the identified variance is below the 50% threshold assessment (Podsakoff et al., 2012). This recommends the absence of common method variance in our data. The single factor Harman's test faced criticism (Chang et al., 2010); hence, we also applied the approach of Liang et al. (2007) approach. First, we calculated the substantive loadings and their square for all the items; then, we introduced a common method factor to the research design. After the inclusion of the common method factor, we analyzed the mentioned once again. The comparison of two analyses revealed that the average squared substantive loadings (0.67%) was more than the squared method loadings (0.08%), as shown in Supplementary Table 2. The insignificant and small loadings of the common method recommend that CMV is not an issue for our data.

### Reliability and Validity

We analyzed the proposed model simultaneously in two steps: analysis of, first, the measurement model and, then, the structural model. An exploratory factor analysis was performed on the 37 items to reveal the underlying patterns of the responses of the participants. Initially, a seven-factor logical solution (with 21 items) was attained with a Kaiser Meyer Olkin test (KMO) .921 and a significant value of Barlet of Sphericity (χ^2^ = 20307, *p* = 0.000). A first-order confirmatory factor analysis (CFA) with the maximum likelihood method was performed. However, our proposed model consists of four first-order and one second-order variable; hence, a CFA for the second-order (risk perception) factor was performed. The result indicates that loadings of all the items are >0.5, as shown in Table 2, which is acceptable (Chen and Tsai, 2007). Besides, composite reliability (CR) was >0.70, Cronbach's alpha (CA) was >0.70, and average variance extracted (AVE) was >0.50, which is on higher side than recommended (Bagozzi et al., 1991; Baer et al., 2008). The results show that all the constructs have discriminant validity as AVE is greater than maximum share variance (MSV), AVE > MSV, as shown in Table 2. Besides, the square root of AVE is greater than the intercorrelation between the constructs, as shown in Table 3 (Fornell and Larcker, 1981; Bagozzi et al., 1991). Discriminant validity means the ability to distinguish between the two constructs. It indicates that the respondents are considering the two constructs as distinct (Sarstedt et al., 2019). These results support the proposed model, as the measurement model has convergent and discriminant validity, composite reliability, and internal scale consistency.

**Table 2:** Confirmatory factor analysis.

**Constructs**	**Items**	**Loading**	**CA**	**CR**	**AVE**	**MSV**
Media Communication (MC)	MC6	0.820	0.912	0.914	0.780	0.444
	MC7	0.888				
	MC8	0.937				
Interpersonal communication (PC)	PC5	0.826	0.885	0.891	0.804	0.384
	PC7	0.962				
Knowledge (KE)	KE4	0.627	0.919	0.925	0.716	0.599
	KE5	0.783				
	KE6	0.911				
	KE7	0.916				
	KE8	0.951				
Travel behavior intentions (TB)	TB1	0.752	0.847	0.854	0.663	0.599
	TB2	0.902				
	TB5	0.780				
Risk Perception (second-order) (RP)	PE	0.824	0.814	0.833	0.624	0.596
	PA	0.753				
	PS	0.796				

**Table 3:** Mean, SD, and correlations.

**Variables**	**Mean**	**SD**	**CR**	**AVE**	**MSV**	**KC**	**MC**	**PC**	**TB**	**RP**
KE	4.156	0.623	0.925	0.716	0.599	**0.846**				
MC	3.957	0.468	0.913	0.779	0.444	0.558	**0.883**			
PC	4.150	0.566	0.891	0.804	0.384	0.511	0.620	**0.897**		
TB	3.782	0.673	0.854	0.662	0.599	0.774	0.556	0.567	**0.814**	
RP	4.234	0.574	0.833	0.624	0.596	0.772	0.666	0.557	0.750	**0.790**

*The bold values are the square root of AVE, It is also called diagonal correlations*.

### The Measurement Model

We analyzed the measurement model MM1 fit with different types of criteria, including the absolute fit, the incremental fit, and the parsimonious fit suggested by Hair et al. (2017). We tested five different measurement models, as shown in Table 4. The measurement model with one second-order and four first-order constructs results in the confirmed fit criteria as the values of the fit indices are within the threshold proposed by Hu and Bentler (1999). The fit indices evidence a good fit for the measurement model as (χ^2^/DF = 5.13, Comparative Fit Index (CFI) = 0.964, Normed Fit Index (NFI) = 0.956, Tucker Lewis Index (TLI) = 0.957, IFI = 0.964, Root Mean Square Error of Approximation (RMSEA) = 0.042, Standardized Root Mean Square Residual (SRMR) = 0.0417), as shown in Table 4. The measurement model MM2 is performed with three first-order and one second-order construct; the χ^2^/DF = 12.42 and other fit indices suggest poor fits. Similarly, MM3 consists of two first-order and one secord-order constructs, MM4 includes two constructs, and MM5 comprises only one construct. The fit indices suggest a poor fit for MM3, MM4, and MM5 (as shown in Table 4), and our data best suit measurement model MM1.

**Table 4:** Measurement and structural model comparison.

**Model**	**Absolute fit**	**SMR**	**RMSEA**	**PCLOSE**	**Incremental fit**	**PNFI**	**Parsimonious fit**	**IFI**	**TLI**
	**χ^2^/DF**				**NFI**		**CFI**		
MM1	5.130	0.0417	0.042	0.000	0.956	0.801	0.964	0.964	0.957
MM2	12.426	0.0508	0.097	0.000	0.907	0.733	0.913	0.913	0.893
MM3	20.779	0.0894	0.128	0.000	0.838	0.703	0.844	0.845	0.814
MM4	26.498	0.0740	0.145	0.000	0.790	0.674	0.796	0.796	0.761
MM5	27.235	0.0781	0.147	0.000	0.782	0.673	0.788	0,788	0.745

*MM2 merges KE and PC, MM3 merges KE, PC, and MC, MM4 merges KE, PC, MC, and TB, MM merges KE, PC, MC, TB, and RP*.

### The Structural Model

This study investigates the association between risk perception, media communication, interpersonal communication, risk knowledge, and travel behavior intentions of Chinese travelers. A structural equation model (SEM) was conducted with a maximum likelihood approach as suggested by Hair et al. (2017). The results indicate that the structural model has a good fit and acceptable, as the fit indices (χ^2^/DF = 5.67, CFI = 0.947, Goodness of Fit Index (GFI) = 0.910, NFI = 0.9939, TLI = 0.938, IFI = 0.947, RMSEA = 0.070, and SRMR = 0.0625) are within the defined threshold recommended by Hu and Bentler (1999).

### Hypotheses Testing

This research tests the proposed hypotheses in two steps: first, perceived severity, perceived anxiety, and perceived efficacy with risk perception; second, the association of risk perception with media communication, interpersonal communication, knowledge, and travel behavior intentions. All the paths were analyzed with standardized coefficients, *t*-values (C.R. = critical ratio), and *p*-values by using Amos 24, as shown in Table 5.

**Table 5:** Path analysis.

**Path**	**Standard coefficient**	***t*-value**	***P*-value**	**Hypotheses**	**Remarks**
COVID-19 risk perception has a positive association with mass media communication	0.68	12.91	0.000	H1a	Supported
COVID-19 risk perception has a positive association with interpersonal communication	0.29	6.065	0.000	H1b	Supported
Media communication has a positive association with risk knowledge	0.41	11.41	0.000	H2a	Supported
Interpersonal communication has a positive association with risk knowledge	0.27	7.833	0.000	H2b	Supported
Media communication has a positive association with interpersonal communication.	0.43	10.18	0.000	H2c	Supported
Risk knowledge has a positive association with travel behavior intentions	0.61	17.83	0.000	H3a	Supported
Media communication has a positive association with travel behavior intentions	0.09	2.953	0.003	H3b	Supported
Interpersonal communication has a positive association with travel behavior intentions	0.20	6.664	0.000	H3c	Supported

*The p-value of “0.000” is just due to a technical approximation, but it's really p < 0.001*.

**Figure 2:**
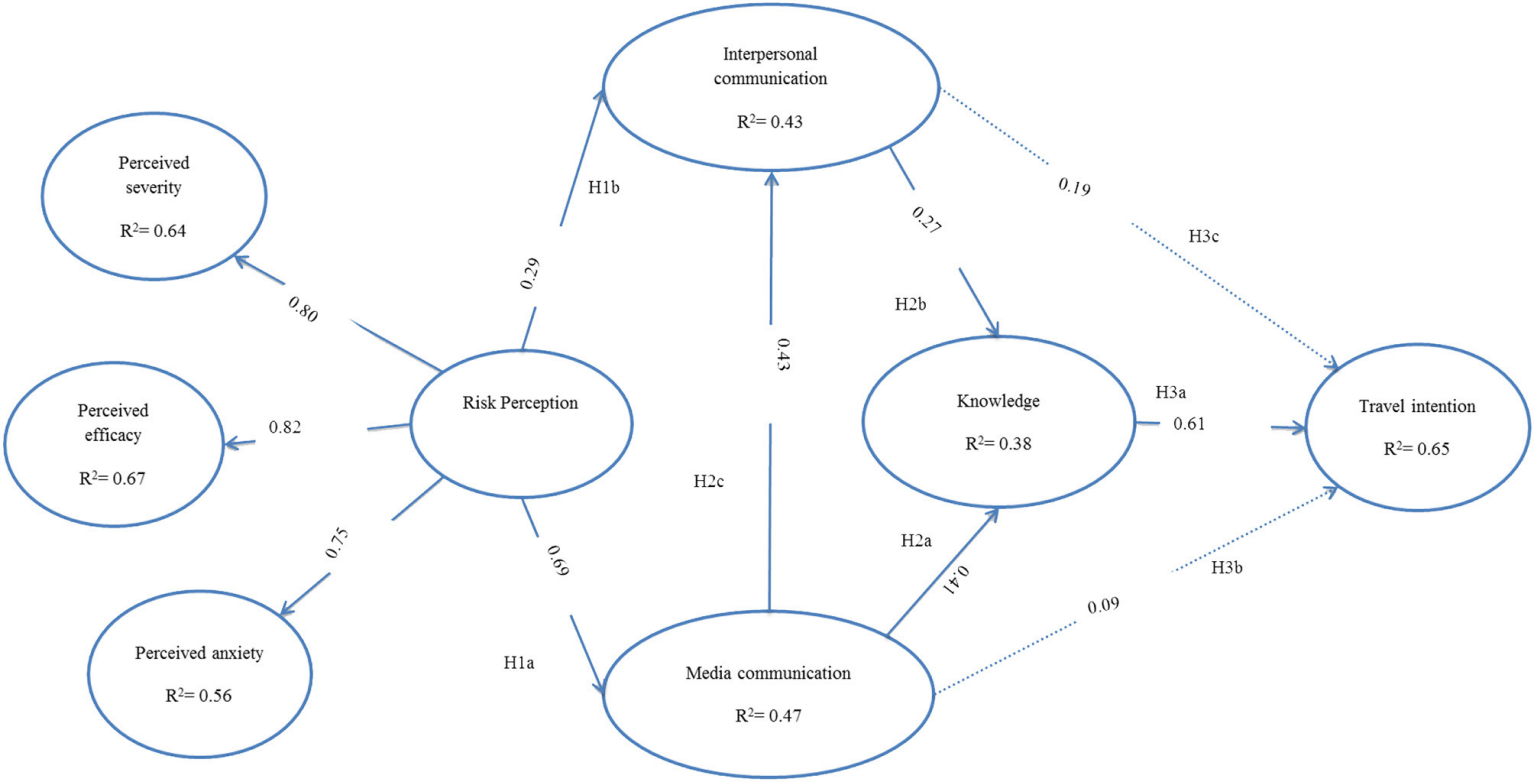
A structural equation model and path analysis.

The results showed that COVID-19 risk perception has a strong positive relationship with perceived severity (β =0.79, *t* = 19.59, *p* < 0.001). The results supported that COVID-19 risk perception has a significant positive association with perceived efficacy (β = 0.82, *t* = 19.66, p < 0.001). The COVID-19 risk perception revealed a significant connection with perceived anxiety (β = 0.754, *t* = 15.51, *p* < 0.001). Risk perception exhibited significant positive linkages with media communication (β =0.69, *t* = 12.91, *p* < 0.001) and, hence, supported H1a. Risk perception also showed a significant positive connection with interpersonal communication (β =0.29, *t* = 6.065, *p* < 0.001) and, hence, supported H1b. The results further indicated that media and interpersonal communication have strong positive linkages with knowledge (β =0.41, *t* = 11.41, *p* < 0.001; and, respectively, β = 0.27 *t* = 7.833, *p* < 0.001) and, hence, supported H2a and H2b. Media communication also exhibited a significant encouraging bond with interpersonal communication (β = 0.43, *t* = 11.41, *p* < 0.001) and, hence, supported H2c. The risk knowledge presented an important positive connection with travel intention (β =0.61, *t* = 17.83, *p* < 0.001) and, hence, supported H3a. Both media and interpersonal communication revealed significant association with travel intention (β = 0.09, *t* = 2.953, *p* =0.003; and, respectively, β =0.20 *t* = 6.664, *p* = *p* < 0.001), hence validating H3b and H3c. All the paths are displayed in Figure 2.

### Moderation Analysis

**Figure 3:**
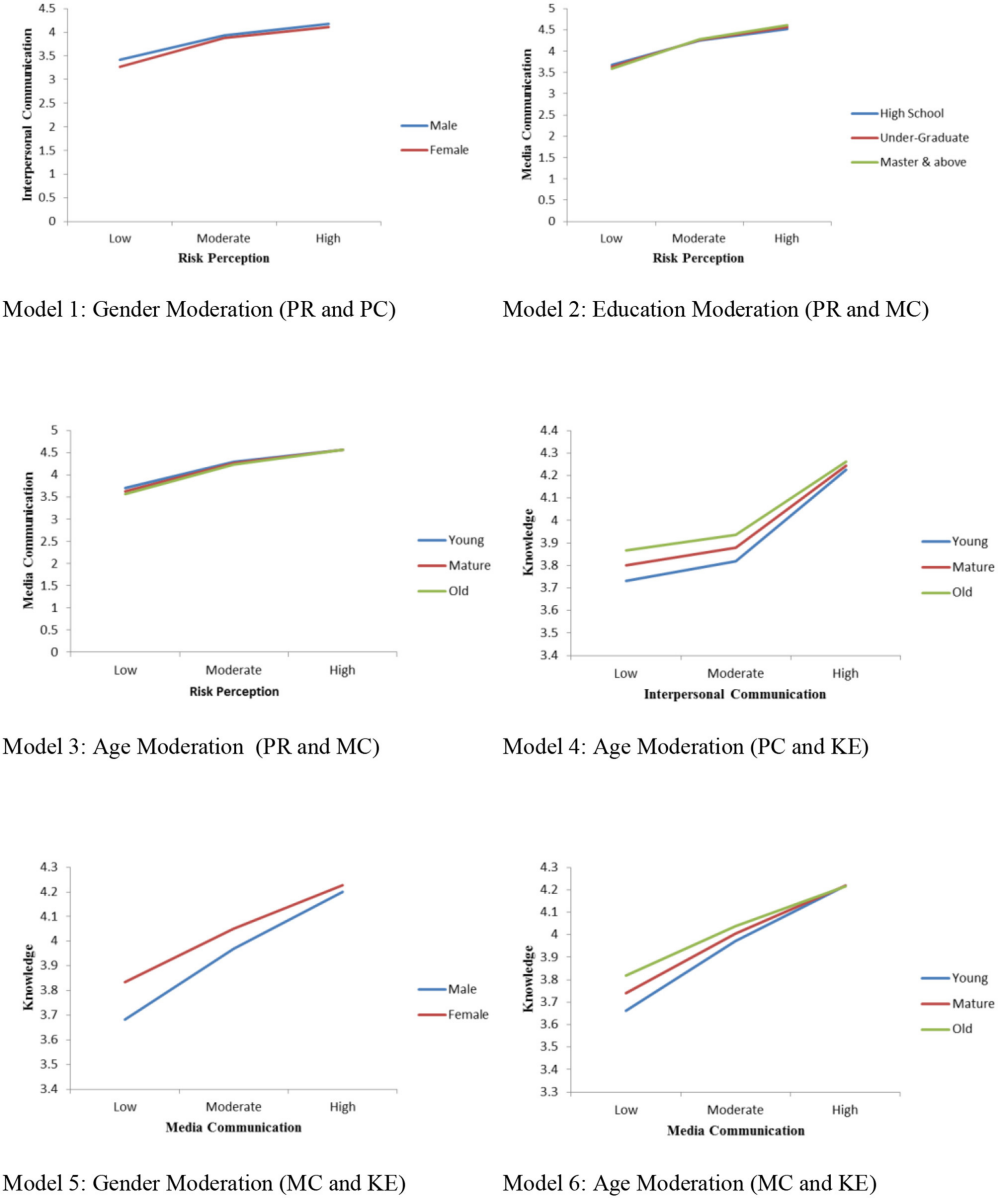
Moderation plots.

We performed SEM and moderation analysis separately. We are interested in knowing whether demographic factors (gender, age, and education) can potentially influence the relationship between risk perception, communication channels, risk knowledge, and travel intentions. Therefore, simple moderation analysis is enough to inform our understandings instead of any other moderation analysis. For instance, Table 6, model 1, consists of two exogenous variables [risk perception (RP) and gender] and one endogenous/dependent variable, personal communication (PC). Using “Andrew Hayes SPSS process macro 3.1” (Hayes, 2017), several moderation analyses were performed to examine whether various demographic factors play any significant role in the relationship between media communication, interpersonal communication, and risk knowledge. The results in Table 6 recommend that gender moderates the relationship between risk perception and interpersonal communication with β = 0.27 *t* = 2.58, *p* < 0.009 and with an overall model fit (R^2^ = 0.39, *F* = 263.82, *p* < 0.001); see model 1 in Table 6; hence, it supported hypothesis H4a. It was also noted that gender moderates the relationship between media communication and knowledge with β = −0.13 *t* = −3.45, *p* < 0.001 and with an overall model fit (*R*^2^ = 0.36, *F* = 227.95, *p* < 0.001); see model 5 in Table 6; thus, it supported hypothesis H4d. Besides, education moderated the relationship between risk perception and media communication β = 0.17 *t* = 3.98, *p* < 0.002 and with an overall model fit (*R*^2^ = 0.55, *F* = 492.21, *p* < 0.001); hence, it supported hypothesis H4b (see, model 2 in Table 6). It was noted that age moderates the relationships between risk perception and media communication with β = 0.11, *t* = 2.85, *p* < 0.004 and with an overall model fit (*R*^2^ = 0.55, *F* = 497.87, *p* < 0.001); therefore, it supported hypothesis H4b (see model 3 in Table 6). Age also moderated the relationship between interpersonal communication and knowledge β = −0.047, *t* = −2.69, *p* < 0.007 and with an overall model fit (*R*^2^ =0.31, *F* = 177.63, *p* < 0.001); thus, it supported hypothesis H4c, and media communication and knowledge β = −0.038, *t* = −4.86, *p* < 0.001 and with an overall model fit (*R*^2^ = 0.36, *F* = 235.28, *p* < 0.001); therefore, it supported hypothesis H4d (see Models 4 and 6 in Table 6). All the plots of the effects of moderation are given in Figure 3.

**Table 6:** Moderation analysis.

				**Bootstrapping**		
**Models**	**Coefficient**	**SE**	**t**	**Significance(p)**	**LLCI**	**ULCI**
**Moderation Model 1 (dependent PC)**						
RP	1.0539	0.1564	6.7371	0.0000	0.7964	1.3115
Gender	−0.7408	0.2621	−2.8270	0.0048	−1.1722	−0.3094
RP × Gender	0.2687	0.1040	2.5820	0.0090	0.0974	0.4399
Conditional Effects						
Male	1.3226	0.0674	19.609	0.0000	1.2116	1.4336
Female	1.5913	0.0792	20.084	0.0000	1.4608	1.7217
**Moderation Model 2 (dependent MC)**						
RP	1.2876	0.1176	10.946	0.0000	1.0940	1.4812
Education	−0.4180	0.1398	−2.9894	0.0029	−0.6482	−0.1878
RP × Education	0.1679	0.0563	3.9838	0.0029	0.0753	0.2606
Conditional Effects						
High school	1.4556	0.0683	21.317	0.0000	1.3432	15680
Under-Graduate	1.6235	0.0427	38.005	0.0000	1.5532	1.6938
Master and above	1.7914	0.0730	24.553	0.0000	1.6713	1.9116
**Moderation Model 3 (dependent MC)**						
RP	1.3874	0.1278	10.074	0.0000	1.0770	1.4977
Age	−0.3181	0.1007	−3.1559	0.0016	−0.4838	−0.1524
Age × PR	0.1141	0.0401	2.8474	0.0045	0.0481	0.1800
Conditional Effects						
Young	1.5155	0.0586	25.866	0.0000	1.4191	1.6120
Mature	1.6266	0.0424	38.433	0.0000	1.5598	1.6994
Old	1.7436	0.0581	30.026	0.0000	1.6480	1.8392
**Moderation Model 4 (dependent KE)**						
PC	0.5676	0.0585	9.6979	0.0000	0.4712	0.6640
Age	0.2306	0.0664	3.4747	0.0005	0.1214	0.3399
PC × Age	−0.0468	0.0173	−2.6977	0.0071	−0.0753	−0.0182
Conditional Effects						
Young	0.4741	0.0280	16.932	0.0000	0.4280	0.5202
Mature	0.4274	0.0191	22.393	0.0000	0.3959	0.4588
Old	0.3806	0.0233	16.302	0.0000	0.3422	0.4190
**Moderation Model 5 (dependent KE)**						
MC	0.6655	0.0588	11.318	0.0000	0.5687	0.7623
Gender	0.6382	0.1516	4.0596	0.0001	0.3794	0.8970
MC × Gender	−0.1301	0.0376	−3.4577	0.0006	−0.1920	−0.0682
Conditional Effects						
Male	0.5355	0.0261	20.521	0.0000	0.4925	0.5784
Female	0.4054	0.0271	14.959	0.0000	0.3608	0.4500
**Moderation Model 6 (dependent KE)**						
MC	0.7460	0.0587	12.699	0.0000	0.6493	0.8427
Age	0.3903	0.0711	5.4879	0.0000	0.2732	0.5073
MC × Age	−0.0833	0.0171	−4.8602	0.0000	−0.1115	−0.0551
Conditional effects						
Young	0.5794	0.0284	20.402	0.0000	0.5326	0.6261
Mature	0.4960	0.0191	25.931	0.0000	0.4646	0.5275
Old	0.4127	0.0227	18.216	0.0000	0.3754	0.4500

## Discussion, Implications, and Limitations

### Discussion of Key Findings

Risk perception is always a central issue in travel and tourism literature (Reisinger and Mavondo, 2005; Wolff et al., 2019). However, minimum attention has been paid to the risk of infectious diseases and their impacts on traveling behavioral intentions. Health safety becomes an essential segment in tourism studies. This study explained individual travel behavioral intention during the COVID-19 outbreak and identified the crucial elements in travel decision-making. There is little known about the public risk perception of infectious diseases as compared with the other domains of risk, such as terrorism, environment, and social conflict. Most of the risk perception of contagious diseases information comes from previous pandemic studies, such as the study by Brug et al. (2004) on SARS risk perception and knowledge, by Gee and Skovdal (2017) on Ebola risk perception, and by Kim et al. (2015) on the H1N1 influenza pandemic risk perception and preventative behaviors.

Although these studies provide useful information, they are descriptive and do not rely on established theories; and they failed to establish the reliability and validity of scales. In contrast, our study adopted a firm theoretical ground, and we established the validity and reliability of the scales for COVID-19. This study is one of the few to examine the underlying mechanism between health risk perception and behavioral intentions of travelers. The confirmation of all hypothesized relationships opens new ways for future work to investigate additional influences within the recommended framework of risk perception and travel intentions. The proposed estimated model recommended that all the factors explained 66% variance in travel behavior intentions.

The risk perception variable used in this research supports the most extensive empirical and theoretical evidence that it is a combination of various sub-constructs (Leppin and Aro, 2009; Bults et al., 2011; Kim et al., 2015). It is found that risk perception explained perceived efficacy more than perceived anxiety and perceived severity. During the COVID-19 outbreak, people are more concerned about taking safety measures and are more concerned about the seriousness of the outbreak, such as getting infected. However, perceived anxiety has a relatively low relationship with risk perception as compared with efficacy and severity in the COVID-19 context. We contradict Cahyanto et al. (2016), who claimed that the Ebola outbreak has minimal effects on traveling behavior because the US government responds quickly to the situation. The magnitude of COVID-19 is much larger than any infectious disease in history; people are more concerned about their health and self-protection. Using protection motivation theory, Wang et al. (2019) attempted to identify self-protective behavior of travelers; however, the study lacks which factor is considered the most by the travelers during an infectious disease outbreak.

This study followed that of Griffin et al. (1999) in explaining the relationship between risk perception and information-seeking behavior through media and interpersonal communication to enhance the knowledge of an individual about the related risk of COVID-19. The path analysis results indicated that risk perception positively influences media and interpersonal communication; this recommended that both communication channels amplify knowledge of an individual. The travelers actively seek information from media channels, and it also helps in initiating interpersonal communication. This research clarified that media communication starts the process of risk information social diffusion and facilitates the amplification of information, as discussed by Kusumi et al. (2017). Both media and interpersonal communication influence risk knowledge; however, media communication adds more weight to risk knowledge as compared with interpersonal communication. Wang et al. (2019) discussed the traveler information-seeking behavior; however, the study lacks details about which source of information influences the traveler behavior the most. In light of social contagion theory, we identified that travelers put more weight on social group information when traveling to a destination.

The path analysis revealed that risk knowledge has a positive association with travel intention. Furthermore, interpersonal communication has a stronger relationship with traveling behavior as compared with media communication, thus providing strong support to contagion theory, which recommends that social network influences the decision-making of individuals. The findings of this study contradicted Snyder and Rouse (1995), who found that media has more impact on behavior than interpersonal communication. Furthermore, demographic factors moderate the relationship between risk perception and other variables, as indicated by the results in Table 6. In conclusion, we acknowledged Schmierer and Jackson (2006), Beirman (2006), Cooper (2006), and Yates (2006), who suggested that risk perception of infectious diseases influences behavioral intentions of travelers; however, their models lack the underlying mechanism of how intentions of travelers are influenced. The current study explained the underlying ruling mechanisms empirically, which are lacking in the literature.

### Theoretical Implications

This study advanced the literature on the mechanism of travel-related risk perception by making various theoretical contributions. This study provides an empirically verified conceptual framework that demonstrates traveler behavioral intentions to risk perception, risk communication, and knowledge building. The crisis and risk literature in tourism has failed to address a common mechanism that illustrates how risk perception forces travelers to search for information to build knowledge about the prevailing risk and then decide about traveling (Carlsen and Liburd, 2008). This study is significant because it provides the researchers with a theoretical foundation to conduct future research related to traveler health risk perception. This study applied a new paradigm to risk perception and traveler behavioral intentions in the context of infectious diseases, which expand the use of risk perception, risk communication, and contagion theories that have been, so far, limited to traveling research (Mileti and Fitzpatrick, 1992; Faulkner, 2001; Fediuk et al., 2010). The conducted model suggests that travel risk is a multidimensional construct and better explains traveler risk perception and intention to travel as indicated by others (Reisinger and Mavondo, 2005); the study contributes to the conceptual validity of risk theories in the travel and tourism context.

COVID-19 matches all the features that influence risk conception suggested by the literature (Slovicj, 1987), such as fatal consequences and unknown uncontrollability. The empirical modeling of the present study explains the underlying mechanism of knowledge building about travel risk and its impact on travel intentions during the outbreak of infectious diseases, hence providing an empirical foundation to the previous risk communication studies (Smith, 2006). During a health risk crisis, travel-related decisions are complicated; therefore, travelers do not only use their risk perceptions but are motivated to search for more information (Quintal et al., 2010). The findings suggested that travelers use media and interpersonal communication to enhance their knowledge and decide about travel. This study proposes that individual travel intention is based on herd behavior (McInnes, 2005).

Thus, individual risk perception and travel intention are sensitive to new information and can easily be changed (Bikhchandani and Sharma, 2000). For instance, the large-scale reduction in tourism and travel activities during SAAR has resulted from people making similar decisions explaining contagion effects. This study contradicts Smith (2006), who proposed that people avoid considering contagions when taking travel actions during SAAR. This study supports the contagion theory (Muter et al., 2013) and risk communication (Aliperti and Cruz, 2018) theories. Research recommends that it is difficult for people to assess the genuine threat of disease (Sandman, 1993) and, hence, search for information. The marked psychological perceived risk and intention to travel during COVID-19 can be attributed to two media and interpersonal information sets.

### Practical Implications

The COVID-19 pandemic disrupted travel and travel planning worldwide. This research investigated the risk perception of COVID-19 among Chinese leisure travelers and its impacts on their future travel intention. The outbound market of China is the largest worldwide, with 150 million international trips being made by the Chinese in 2018 (UNWTO, 2019). The COVID-19 risk perception limited travelers to stay close to home; hence, if the restrictions on inbound and outbound travel are lifted, people will still have concerns about traveling.

The findings suggest that travelers are more inclined toward self-protection as the efficacy coefficient is higher than the anxiety and severity coefficient. Thus, travel and tourism-related businesses are required to focus high on hygiene and cleanness to reassure travelers that they are safe. Touch-free service will become a necessity for consumers (e.g., touch-free transactions and touch-free deliveries) to avoid catching the virus when they travel (Khan et al., 2020d). Contactless technologies, such as Ali Pay, delivery drones, and robots, that have struggled for adoption will get a new push. Tourist sites should require advanced bookings to limit crowds. Some have been told to limit guests to 30% of capacity amid lingering COVID-19 worries. The moderation indicated that low-educated travelers are less concerned about risk information as compared with highly educated; thus, travel marketing professionals, destination, and attractions managers should focus their marketing toward less-educated travelers class immediately after the pandemic. The gender moderation effect of risk perception and interpersonal communication revealed that women are less sensitive to seeking risk information in low- to medium-risk situations, indicating that women are more risk resilient than men. Travel and tourism organizations should focus more on female Chinese travelers for bookings/reservation during and after the pandemic. The moderation effects of age between risk perception and media information-seeking behavior revealed that young and mature people are inclined to seek media risk information during the low-risk perception stage, and older people are more motivated to find media risk information during the high-risk perception stage. Hence, older people are more concerned when risk severity is high and have a high level of travel anxiety. Thus, marketing campaigns in high-risk situations should focus on young travelers, and older people should be the target in low-risk situations.

The association between communication channels and risk knowledge-building scenarios during the pandemic era offers a sustainable communication layout to travel and tourism organizations. They should focus on virtual tours to keep travelers engaged and motivated. Live streaming events can be used as an engagement tool for the museums, theatres, and scenic spots. They will offer an experience that can be enjoyed while adhering to travel restrictions. The situation of social distancing with COVID-19 offers the rural destinations an opportunity to focus on marketing where social distance is not an issue. Crisis and opportunities often go together; however, each major event has raised business opportunities. Despite the impact of the outbreak, it could accelerate developments in the industry in several ways: First, by driving the rearrangement of traditional tourism and the refreshing of new tourism models. The industry should focus on customer needs, customize its products, optimize product expressions, refine operations, and establish long-term relationships with users.

Service providers also need to stay in touch with customers, thereby securing customer loyalty and maintaining a keen sense of their needs; second, by advancing the digitalization of the tourism industry. Online short video marketing has received a lot of attention during the epidemic, and offline tourism operators could now consider reaching users through short video interactive projects. It will keep people interested in travel. It is advised that travel and tourism organizations continue advertisements to hold themselves on the minds of the traveler. Destination management organizations should focus on motivational marketing campaigns, such as “Till Then, Stay Safe,” to urge international and domestic travelers to stay safe during the pandemic and continuing dreaming and planning to escape their stunning beautiful destinations. This short communication strategy will keep the destinations on top of the minds of travelers and make them feel worthy for the destinations.

The proposed model of this study also suggests the communication mechanism for the revival of the traveling industry. As travelers seek risk information through mass media and interpersonal communication that influence their traveling behavior, a reverse communication strategy with the same channels could be used during and after the pandemic to change their perception that they are safe during their travel and at the destinations. To attract and regain the trust of existing and prospective customers, travel and tourism organizations need to communicate aggressively to the customers through the mass media at large and social media at the individual level. Tourism destinations, hotels, airlines, and travel agencies should use Facebook, Twitter, Instagram, and Zoom to connect people from different cultures and natural worlds. Resources like webcams in national parks, free virtual museums, Google arts, and virtual culture films offer a potential substitute experience that might help engage and motivate tourists for future travel. In this environment of travel restrictions and fear, it is essential for travel and tourism professionals to communicate marketing messages with the right tone; for instance, messages should be respectful and sensitive to the current situation. Most credible sources should be identified to present your story (destinations, travel organizations, airlines, and hotels) to the audience backed by visible actions and audiovisual responses to the crisis. During and after the pandemic, there are opportunities to build brand equity through media relations; for instance, the relationship of travel journalists is an appetite to earn media space in a crisis.

Besides, the COVID-19 impact will be felt for some time. It is now more important than ever for all the major stakeholders of the tourism and hospitality industry (airports, airlines, transport, hotels, destinations, natural and theme parks) to prepare for the new reality. All the stakeholders should prioritize health and safety commitment to deliver a queueless, contactless, and sanitized end-to-end travel experience that is automated as much as possible. Thus, technology remains a key option for reviving travel; electronic IDs, passports, boarding passes, robot cleaners, and medical screening should be deployed to minimize physical contact between travelers and the surface. In the short run, it is not wise to think about starting international traveling due to the second outbreak of COVID-19; thus, to support the tourism and hospitality industry, travel companies should be focused first to encourage domestic tourism. Meanwhile, domestic tourism and travel are expected to be a substitute for foreign tourism demand.

### Limitations and Future Research Directions

This study provides a solid theoretical and empirical ground for the researcher to apply these approaches to longitudinal studies to study the impact of the global pandemic on travel intentions. We have focused on Chinese leisure travelers; new research should address various nationalities and travelers from different social and cultural backgrounds to discover their use of communication channels for acquiring risk knowledge about pandemics and use of this knowledge to decide about traveling. Besides, identifying risk-searching information behavior in diverse tourism and traveling settings is important for formulating marketing strategies to modify travel intention accordingly. This study is based on a cross-sectional survey design, which is its main limitation; besides, it has been collected only from Chinese leisure travelers. The findings reveal that international travelers are more concerned about their safety. The severity of the disease contributes more to the risk perception than anxiety. Media communication contributes higher to risk knowledge building than interpersonal communication. However, interpersonal communication is more vital than media communication in travel decision-making. It shows that people pay more attention to their family, friends, and near circles in making decisions. This study applied the snowball sampling technique, which constrains the findings. We used this sampling technique because of limited access to the participants due to the high risk of COVID-19. Each participant was asked to forward the questionnaire to his/her peer group members (family, friends, colleagues who have traveled abroad within the past 3–5 years). This sampling technique is criticized for sample representativeness. Hence, our sample may be some issues of representativeness. However, our study provides possible strategic implications and informs tourism and hospitality organizations what segments of the market they can target.

## Data Availability Statement

The raw data supporting the conclusions of this article will be made available by the authors, without undue reservation.

## Author Contributions

AK and SB contributed equally to the conception, study design, and writing of the original manuscript. YM helped in the idea generation. WC revised the manuscript. YL helped in data collection and literature collection. All authors contributed to the article and approved the submitted version.

## Conflict of Interest

The authors declare that the research was conducted in the absence of any commercial or financial relationships that could be construed as a potential conflict of interest.
