# Supplementary material for: The Effects of COVID-19 Risk Perception on Travel Intention: Evidence From Chinese Travelers
Source: Front Psychol. 2021 Jul 16;12:655860. doi: 10.3389/fpsyg.2021.655860 (PMC8322978; doi:10.3389/fpsyg.2021.655860)
Supplement: Supplementary file 1 [file Data_Sheet_1.docx]

**Supplementary Material for different parts of the article**

***Theoretical framework***

Mass communication is a significant determinant of individual information (Kasperson et al., 1988; Kusumi, Hirayama, & Kashima, 2017). The mass media initiates the process of social diffusion of risk information, hence facilitating amplification or attenuating the public risk perception knowledge (Binder, 2010; Coleman, 1993; Kusumi et al., 2017). Studies suggest that interpersonal networks influence the adoption of ideas, risk perception, and behavioral intentions.

***Risk perception***

Risk is often taken as the cognitive evaluation of an outcome's probability and severity (Slovic, Kunreuther, & White, 2016). Risk is also defined as exposure to dangers or threats (Reisinger & Mavondo, 2005; Williams & Baláž, 2013). Since perceived risk is seen in terms of probable loss, hence, scholars suggested that perceived risk is a combination of various potential losses (Dholakia, 2001; Jacoby & Kaplan, 1972). Risk perception cannot be directly observable and measurable as it is a multidimensional variable (Cunningham, 1967; Jacoby & Kaplan, 1972).

***Risk perception of Covid-19 and intention to travel***

The demand and need for travel have been extensively documented in tourism literature (Law, 2006). People travel for business, leisure, visit friends, and relatives (VFR), vacations, and several reasons (Williams & Baláž, 2015). Rational travelers avoid taking a risk that may threaten their health and safety (Rittichainuwat & Chakraborty, 2009). In March 2003, inbound tourist arrival to Thailand significantly decreased due to SARS outbreak (Rittichainuwat & Chakraborty, 2009). Risk theories suggest that tourist intention to travel is determined by risk beliefs consist of perceived anxiety, efficacy, and the severity of risk (J. Wang, Liu-Lastres, Ritchie, & Pan, 2019). Lee, Song, Bendle, Kim, and Han (2012) noted that the HINI influenza pandemic discourages international traveling. Hall (2006) noted that climate changes and infectious diseases are changing the tourism industry. The speedy spread of SARS and the World Health Organization (WHO) advisories caused a vivid reduction in tourist arrivals in 2003 (Yates, 2006). McKercher and Chon (2004) noted that 70% or more tourist arrivals fall across the Asian region due to SARS and severely affected China, Hong Kong, and Singapore economies. Schmierer and Jackson (2006) suggested that the perceived risk of infectious disease highly affects tourist behavior; for instance, SARS' perceived risk changes travelers' behavior to avoid traveling (Beirman, 2006; Cooper, 2006). Travelers avoided traveling to the SARS originating countries and the neighboring nations where no SARS case was reported (Cooper, 2006; McKercher & Chon, 2004; Yates, 2006).

In travel intention and health behavior theories, perceived risk is presented as a central construct referred to perceived likelihood, probability, and susceptibility (Brewer, Weinstein, Cuite, & Herrington, 2004; Chien, Sharifpour, Ritchie, & Watson, 2017). Roehl and Fesenmaier (1992) researched the relationship between leisure travel and perceived risk and found that travel behavior is situation-specific. Travel risk perception is the negative valenced likelihood assessment of an unfavorable event that will occur at a specific time (Menon, Raghubir, & Agrawal, 2006). The recent Ebola virus disease epidemic suggested a devasting decrease in tourist arrivals to African countries (Novelli, Burgess, Jones, & Ritchie, 2018). With few exceptional situations, most of the literature are agreed that travelers avoid traveling during a pandemic, epidemic, and health riskier destinations (Boksberger, Bieger, & Laesser, 2007; Cooper, 2006; Page, Yeoman, Munro, Connell, & Walker, 2006; Rittichainuwat & Chakraborty, 2009; J. Wang, Liu-Lastres, Ritchie, & Mills, 2019). The theories of risk, such as the theory of reasoned behavior actions (TRA) and planned behavior, suggest that travelers' perceived risk is positively associated with intention to avoid traveling; in other words, perceived risk has a negative relationship with intention to travel (Casaló, Flavián, & Guinalíu, 2010; Griffin, Dunwoody, & Neuwirth, 1999; Lee et al., 2012).

***Interpersonal and media communication***

The research found that interpersonal communication mediates media's influence on human behavior (Katz, 1987). Studies also found that media and interpersonal communication have a convergent relationship (Southwell & Yzer, 2007). The research noted that interpersonal communication has a positive relationship with mass media communication (Dudo, Dahlstrom, & Brossard, 2007; Ho, 2012).

**Research Method**

China's tourism industry is multiplying and becoming a significant part of the Chinese economy (Li, Harrill, Uysal, Burnett, & Zhan, 2010). The domestic and outbound travel boom in China is due to the emergence of an affluent middle class and ease of movement (Huang, Keating, Kriz, & Heung, 2015). Over the last few decades, since the beginning of the reforms and open-door policy, China has become the busiest outbound and inbound tourist market (Shambaugh, 2013). It was estimated that China's number of domestic trips would increase to about 2.38 billion trips by 2020 (Rosen, 2018). “China is the single largest outbound travel market in the world in terms of spending” (Ying, Wang, Liu, Wen, & Goh, 2020). The major factors driving the China outbound tourism market's growth include a rising affluent middle-class population, liberal tourism policy, and an open-door policy. Accordingly, this study was conducted in Mainland China. The motivation behind choosing Mainland China was due to several reasons. First, China offers one of the topmost outbound tourism markets in the world (Huang et al., 2015); second, the first case of the COVID-19 was reported in Wuhan (Dong, Du, & Gardner, 2020); third, the risk perception and knowledge about the COVID-19 spread from China (C. Wang et al., 2020).

**Appendix AI**

| **Risk Perception of COVID-19** | (Brug et al., 2004), (Bults et al., 2011), (Kim, Zhong, Jehn, & Walsh, 2015), (Lau, Yang, Tsui, & Kim, 2003) |
| --- | --- |
| ***Perceived severity*** |  |
| Coronavirus make the situation severe |  |
| The severity of getting Coronavirus is high |  |
| The severity of getting Coronavirus is high |  |
| The Coronavirus is very harmful to my health |  |
| ***Perceived anxiety*** |  |
| Worried about getting the Coronavirus |  |
| Fear for my family may get the Coronavirus |  |
| Thinking about Coronavirus and health problems |  |
| ***Perceived efficacy*** |  |
| Keep away from crowded places |  |
| Practice better hygiene |  |
| Avoid regions/person with sign of infection |  |
| Wear face mask to protect for Coronavirus |  |
| Seek medical advice with the onset of flu, fever, cough symptoms |  |
| Ability to avoid Coronavirus compared to others |  |
| **Media Communication** | (Gao, Li, Ling, Dou, & Liu, 2019), (Gee & Skovdal, 2017), (Luna-Cortés, López-Bonilla, & López-Bonilla, 2019) |
| Reading magazines |  |
| Reading new papers |  |
| Watching TV |  |
| Using the Internet |  |
| Pay attention to government directives |  |
| Media news is the main sources of information that I use in order to plan my trip |  |
| I frequently use media news to decide about my vacations |  |
| Media news has become an important source of information for the decisions I make before I travel |  |
| **Interpersonal Communication** | (Gao et al., 2019), (Gee & Skovdal, 2017), (Luna-Cortés et al., 2019) |
| Meeting family and friends |  |
| Talking to friends and family on wechat |  |
| Visiting neighbors |  |
| Reading friends & family movements |  |
| My social networks are the main sources of information that I use in order to plan my trip |  |
| I frequently use my social network to decide about my vacations |  |
| My social networks have become an important source of information for the decisions I make before I travel |  |
| Uploading information about the experience of daily life in my social network is an integral part of the daily routine |  |
| **Risk Knowledge** | (Brug et al., 2004), (Bults et al., 2011), (Kim et al., 2015), (Gao et al., 2019), (Suratman, Ross, Babina, & Edwards, 2016) |
| Causes of coronavirus |  |
| A vaccine is available against the Coronavirus |  |
| The animal can transmit the Coronavirus to human |  |
| coronavirus can transmit from human to human |  |
| Symptoms of Coronavirus are visible Coronavirus |  |
| Know of school closed due to Coronavirus |  |
| Know of activities closed due to Coronavirus |  |
| Know of businesses closed to Coronavirus |  |
| **Traveling behavior** | (Desivilya, Teitler-Regev, & Shahrabani, 2015), (Schroeder, Pennington-Gray, Kaplanidou, & Zhan, 2013) |
| I feel nervous about traveling right now |  |
| Traveling is risky right now |  |
| Because of Coronavirus, mega-events should be avoided |  |
| I feel like I could experience barriers while traveling internationally |  |
| I would likely experience health-related problems when I travel |  |
| It is preferable to avoid travel during outbreak incidents |  |
| It is preferable to avoid traveling due to health hazards |  |
| *Note: All the items are measured with a five-point Likert scale, where 1 stands for strongly disagreed and 5 for strongly agreed. Risk perception has been measured with three sub-scale including perceived severity, perceived anxiety, and perceived efficacy.* | |

Supplementary Table 2. Common Method Variance

| **Constructs** | **Items** | **Substantive R1** | **R1^2^** | **Common method loading R2** | **R2^2^** |
| --- | --- | --- | --- | --- | --- |
| Perceived Efficacy (PE) | PSA1 | 0.864 | 0.746 | 0.329 | 0.108 |
|  | PSA2 | 0.845 | 0.714 | 0.339 | 0.115 |
|  | PSA3 | 0.850 | 0.723 | 0.330 | 0.109 |
| Perceived Severity (PS) | PS2 | 0.811 | 0.658 | 0.244 | 0.060 |
|  | PS3 | 0.807 | 0.651 | 0.303 | 0.092 |
| Perceived anxiety (PA) | PA1 | 0.768 | 0.590 | 0.288 | 0.083 |
|  | PA2 | 0.796 | 0.634 | 0.245 | 0.060 |
|  | PA3 | 0.699 | 0.489 | 0.249 | 0.062 |
| Media communication (MC) | MC6 | 0.847 | 0.717 | 0.303 | 0.092 |
|  | MC7 | 0.856 | 0.733 | 0.282 | 0.080 |
|  | MC8 | 0.826 | 0.682 | 0.269 | 0.072 |
| Interpersonal communication | PC5 | 0.902 | 0.814 | 0.283 | 0.080 |
|  | PC7 | 0.810 | 0.656 | 0.264 | 0.070 |
| Knowledge | KE4 | 0.627 | 0.393 | 0.352 | 0.124 |
|  | KE5 | 0.783 | 0.613 | 0.326 | 0.106 |
|  | KE6 | 0.911 | 0.830 | 0.254 | 0.065 |
|  | KE7 | 0.916 | 0.839 | 0.221 | 0.049 |
|  | KE8 | 0.951 | 0.904 | 0.334 | 0.112 |
| Travel behavior intentions | TB1 | 0.779 | 0.607 | 0.283 | 0.080 |
|  | TB2 | 0.769 | 0.591 | 0.328 | 0.108 |
|  | TB5 | 0.780 | 0.608 | 0.277 | 0.077 |
| Sum |  | 17.197 | 14.192 | 6.103 | 1.801 |
| Average |  |  | 0.6758 |  | 0.0857 |
| For detailed methodology, please see: (Kanwal, Rasheed, Pitafi, Pitafi, & Ren, 2020). | | | | | |

Beirman, D. (2006). A comparative assessment of three Southeast Asian tourism recovery campaigns: Singapore roars: post SARS 2003, Bali post-the October 12, 2002 bombing, and WOW Philippines 2003. *Tourism, security and safety: From theory to practice*, 251-269.

Binder, A. R. (2010). Routes to attention or shortcuts to apathy? Exploring domain-specific communication pathways and their implications for public perceptions of controversial science. *Science Communication, 32*(3), 383-411.

Boksberger, P. E., Bieger, T., & Laesser, C. (2007). Multidimensional analysis of perceived risk in commercial air travel. *Journal of Air Transport Management, 13*(2), 90-96.

Brewer, N. T., Weinstein, N. D., Cuite, C. L., & Herrington, J. E. (2004). Risk perceptions and their relation to risk behavior. *Annals of behavioral medicine, 27*(2), 125-130.

Brug, J., Aro, A. R., Oenema, A., De Zwart, O., Richardus, J. H., & Bishop, G. D. (2004). SARS risk perception, knowledge, precautions, and information sources, the Netherlands. *Emerging infectious diseases, 10*(8), 1486.

Bults, M., Beaujean, D. J., de Zwart, O., Kok, G., van Empelen, P., van Steenbergen, J. E., . . . Voeten, H. A. (2011). Perceived risk, anxiety, and behavioural responses of the general public during the early phase of the Influenza A (H1N1) pandemic in the Netherlands: results of three consecutive online surveys. *BMC public health, 11*(1), 2.

Casaló, L. V., Flavián, C., & Guinalíu, M. (2010). Determinants of the intention to participate in firm-hosted online travel communities and effects on consumer behavioral intentions. *Tourism Management, 31*(6), 898-911.

Chien, P. M., Sharifpour, M., Ritchie, B. W., & Watson, B. (2017). Travelers’ health risk perceptions and protective behavior: a psychological approach. *Journal of Travel Research, 56*(6), 744-759.

Coleman, C.-L. (1993). The influence of mass media and interpersonal communication on societal and personal risk judgments. *Communication Research, 20*(4), 611-628.

Cooper, M. (2006). Japanese tourism and the SARS epidemic of 2003. *Journal of Travel & Tourism Marketing, 19*(2-3), 117-131.

Cunningham, M. S. (1967). The major dimensions of perceived risk. *Risk taking and information handling in consumer behavior*.

Desivilya, H., Teitler-Regev, S., & Shahrabani, S. (2015). The effects of conflict on risk perception and travelling intention of young tourists. *EuroMed Journal of Business*.

Dholakia, U. M. (2001). A motivational process model of product involvement and consumer risk perception. *European Journal of Marketing*.

Dong, E., Du, H., & Gardner, L. (2020). An interactive web-based dashboard to track COVID-19 in real time. *The Lancet infectious diseases*.

Dudo, A. D., Dahlstrom, M. F., & Brossard, D. (2007). Reporting a potential pandemic: A risk-related assessment of avian influenza coverage in US newspapers. *Science Communication, 28*(4), 429-454.

Gao, S., Li, W., Ling, S., Dou, X., & Liu, X. (2019). An Empirical Study on the Influence Path of Environmental Risk Perception on Behavioral Responses In China. *International journal of environmental research and public health, 16*(16), 2856.

Gee, S., & Skovdal, M. (2017). The role of risk perception in willingness to respond to the 2014–2016 West African Ebola outbreak: a qualitative study of international health care workers. *Global health research and policy, 2*(1), 21.

Griffin, R. J., Dunwoody, S., & Neuwirth, K. (1999). Proposed model of the relationship of risk information seeking and processing to the development of preventive behaviors. *Environmental research, 80*(2), S230-S245.

Hall, C. M. (2006). Tourism, disease and global environmental change: the fourth transition? *Tourism and global environmental change* (pp. 173-193): Routledge.

Ho, S. S. (2012). The knowledge gap hypothesis in Singapore: The roles of socioeconomic status, mass media, and interpersonal discussion on public knowledge of the H1N1 flu pandemic. *Mass Communication and Society, 15*(5), 695-717.

Huang, S., Keating, B. W., Kriz, A., & Heung, V. (2015). Chinese outbound tourism: An epilogue. *Journal of Travel & Tourism Marketing, 32*(1-2), 153-159.

Jacoby, J., & Kaplan, L. B. (1972). The components of perceived risk. *ACR Special Volumes*.

Kanwal, S., Rasheed, M. I., Pitafi, A. H., Pitafi, A., & Ren, M. (2020). Road and transport infrastructure development and community support for tourism: The role of perceived benefits, and community satisfaction. *Tourism Management, 77*, 104014.

Kasperson, R. E., Renn, O., Slovic, P., Brown, H. S., Emel, J., Goble, R., . . . Ratick, S. (1988). The social amplification of risk: A conceptual framework. *Risk analysis, 8*(2), 177-187.

Katz, E. (1987). Communications research since Lazarsfeld. *The Public Opinion Quarterly, 51*, S25-S45.

Kim, Y., Zhong, W., Jehn, M., & Walsh, L. (2015). Public risk perceptions and preventive behaviors during the 2009 H1N1 influenza pandemic. *Disaster medicine and public health preparedness, 9*(2), 145-154.

Kusumi, T., Hirayama, R., & Kashima, Y. (2017). Risk perception and risk talk: The case of the Fukushima Daiichi nuclear radiation risk. *Risk analysis, 37*(12), 2305-2320.

Lau, J., Yang, X., Tsui, H., & Kim, J. (2003). Monitoring community responses to the SARS epidemic in Hong Kong: from day 10 to day 62. *Journal of Epidemiology & Community Health, 57*(11), 864-870.

Law, R. (2006). The perceived impact of risks on travel decisions. *International Journal of Tourism Research, 8*(4), 289-300.

Lee, C.-K., Song, H.-J., Bendle, L. J., Kim, M.-J., & Han, H. (2012). The impact of non-pharmaceutical interventions for 2009 H1N1 influenza on travel intentions: A model of goal-directed behavior. *Tourism Management, 33*(1), 89-99.

Li, X. R., Harrill, R., Uysal, M., Burnett, T., & Zhan, X. (2010). Estimating the size of the Chinese outbound travel market: A demand-side approach. *Tourism Management, 31*(2), 250-259.

Luna-Cortés, G., López-Bonilla, L. M., & López-Bonilla, J. M. (2019). The influence of social value and self-congruity on interpersonal connections in virtual social networks by Gen-Y tourists. *PloS one, 14*(6).

McKercher, B., & Chon, K. (2004). The over-reaction to SARS and the collapse of Asian tourism. *Annals of tourism research, 31*(3), 716-719.

Menon, G., Raghubir, P., & Agrawal, N. (2006). Health risk perceptions and consumer psychology. *Available at SSRN 945673*.

Novelli, M., Burgess, L. G., Jones, A., & Ritchie, B. W. (2018). ‘No Ebola… still doomed’–The Ebola-induced tourism crisis. *Annals of tourism research, 70*, 76-87.

Page, S., Yeoman, I., Munro, C., Connell, J., & Walker, L. (2006). A case study of best practice—Visit Scotland's prepared response to an influenza pandemic. *Tourism Management, 27*(3), 361-393.

Reisinger, Y., & Mavondo, F. (2005). Travel anxiety and intentions to travel internationally: Implications of travel risk perception. *Journal of Travel Research, 43*(3), 212-225.

Rittichainuwat, B. N., & Chakraborty, G. (2009). Perceived travel risks regarding terrorism and disease: The case of Thailand. *Tourism Management, 30*(3), 410-418.

Roehl, W. S., & Fesenmaier, D. R. (1992). Risk perceptions and pleasure travel: An exploratory analysis. *Journal of Travel Research, 30*(4), 17-26.

Rosen, E. (2018). New Rankings Of The World's Fastest-Growing Tourism Destinations. *Forbes*.

Schmierer, C., & Jackson, M. (2006). Local health impacts of tourism *Tourism in Turbulent Times* (pp. 87-100): Routledge.

Schroeder, A., Pennington-Gray, L., Kaplanidou, K., & Zhan, F. (2013). Destination risk perceptions among US residents for London as the host city of the 2012 Summer Olympic Games. *Tourism Management, 38*, 107-119.

Shambaugh, D. L. (2013). *China goes global: The partial power* (Vol. 409): Oxford University Press Oxford.

Slovic, P., Kunreuther, H., & White, G. (2016). Decision processes, rationality and adjustment to natural hazards *The perception of risk* (pp. 39-69): Routledge.

Southwell, B. G., & Yzer, M. C. (2007). The roles of interpersonal communication in mass media campaigns. *Annals of the International Communication Association, 31*(1), 420-462.

Suratman, S., Ross, K. E., Babina, K., & Edwards, J. W. (2016). The effectiveness of an educational intervention to improve knowledge and perceptions for reducing organophosphate pesticide exposure among Indonesian and South Australian migrant farmworkers. *Risk management and healthcare policy, 9*, 1.

Wang, C., Pan, R., Wan, X., Tan, Y., Xu, L., Ho, C. S., & Ho, R. C. (2020). Immediate psychological responses and associated factors during the initial stage of the 2019 coronavirus disease (COVID-19) epidemic among the general population in china. *International journal of environmental research and public health, 17*(5), 1729.

Wang, J., Liu-Lastres, B., Ritchie, B. W., & Mills, D. J. (2019). Travellers' self-protections against health risks: An application of the full Protection Motivation Theory. *Annals of tourism research, 78*, 102743.

Wang, J., Liu-Lastres, B., Ritchie, B. W., & Pan, D.-Z. (2019). Risk reduction and adventure tourism safety: An extension of the risk perception attitude framework (RPAF). *Tourism Management, 74*, 247-257.

Williams, A. M., & Baláž, V. (2013). Tourism, risk tolerance and competences: Travel organization and tourism hazards. *Tourism Management, 35*, 209-221.

Williams, A. M., & Baláž, V. (2015). Tourism risk and uncertainty: Theoretical reflections. *Journal of Travel Research, 54*(3), 271-287.

Yates, M. (2006). Project Phoenix: a benchmark for reputation management in travel and tourism. *Tourism in turbulent times: towards safe experiences for visitors*, 263-276.

Ying, T., Wang, K., Liu, X., Wen, J., & Goh, E. (2020). Rethinking game consumption in tourism: a case of the 2019 novel coronavirus pneumonia outbreak in China. *Tourism Recreation Research*, 1-6.
